# Criterion validity and divergent risk profiles of long-term opioid therapy across medicare and medicaid

**DOI:** 10.1371/journal.pone.0347943

**Published:** 2026-04-29

**Authors:** Robert W. Hurley, Daniel Guth, Elaine L. Hill, Meredith C. B. Adams

**Affiliations:** 1 Department of Anesthesiology, Wake Forest University School of Medicine, Medical Center Boulevard, Winston-Salem, North Carolina, United States of America; 2 Department of Translational Neuroscience, Wake Forest University School of Medicine, Medical Center Boulevard, Winston-Salem, North Carolina, United States of America; 3 Department of Public Health, Wake Forest University School of Medicine, Medical Center Boulevard, Winston-Salem, North Carolina, United States of America; 4 Pain Outcomes Lab, Wake Forest University Health Sciences, Medical Center Boulevard, Winston-Salem, North Carolina, United States of America; 5 Department of Public Health Sciences, University of Rochester Medical Center, Rochester, New York, United States of America; 6 Center for Artificial Intelligence, Wake Forest University School of Medicine, Medical Center Boulevard, Winston-Salem, North Carolina, United States of America; Virginia Commonwealth University School of Medicine, UNITED STATES OF AMERICA

## Abstract

**Importance:**

Accurate identification of patients receiving long-term opioid therapy (LTOT) remains essential for clinical care and population health surveillance. Two widely used methods: prescription-based definitions and diagnostic codes are often treated as interchangeable, yet the criterion validity of code-based identification against a prescription-based reference standard is unknown, and the comparative risk profiles associated with each method across diverse populations are incompletely understood.

**Objective:**

To assess the criterion validity of diagnostic codes for identifying long-term opioid therapy (LTOT) using prescription-based LTOT as the reference standard, and to compare how each identification method corresponds to the risk of opioid use disorder, opioid poisoning, and other adverse outcomes across Medicare and Medicaid populations.

**Design, setting, and participants:**

Retrospective cohort study using 100% Medicare and Medicaid claims data from 2016–2022. Criterion validity was assessed using complete beneficiary populations with a pain diagnosis (65.5 million Medicaid; 59.6 million Medicare). Risk-profile comparisons used incident LTOT cohorts identified by prescription-based 90-day LTOT, Z79.891 code, or both, excluding those with pre-existing opioid use disorder diagnoses.

**Main outcomes and measures:**

Criterion validity metrics included sensitivity, specificity, positive predictive value (PPV), negative predictive value (NPV), and Cohen’s kappa for Z79.891 against prescription-based LTOT. Within incident LTOT cohorts, primary outcomes within 2 years included documented OUD diagnosis, opioid poisoning, medication for OUD (MOUD) receipt, all-cause mortality, drug mortality, and emergency department visits for pain.

**Results:**

In the full Medicaid population (N = 65.5 million), Z79.891 demonstrated sensitivity of 30.5% and specificity of 97.3% against prescription-based LTOT, with PPV of 40.2% and Cohen’s κ = 0.313 (fair agreement). In Medicare (N = 59.6 million), sensitivity was 34.6%, specificity 91.4%, PPV 23.0%, and κ = 0.210 (fair agreement). Approximately two-thirds of prescription-based LTOT patients lacked Z79.891 coding in both populations. Among incident LTOT cohorts (771,581 Medicaid; 4,376,993 Medicare), the two identification methods demonstrated divergent risk profiles across insurance programs. In Medicaid, patients identified through prescription-based LTOT (n = 390,793) had significantly lower odds compared to code-based LTOT patients (n = 267,577) for OUD diagnosis (OR, 0.713; 95% CI, 0.696–0.730) and MOUD receipt (OR, 0.374; 95% CI, 0.364–0.385), but higher odds for all-cause mortality (OR, 2.277; 95% CI, 2.224–2.331) and opioid poisoning (OR, 1.117; 95% CI, 1.063–1.173). In Medicare, this pattern reversed: prescription-based LTOT patients (n = 1,045,466) demonstrated significantly higher odds than code-based LTOT patients (n = 2,845,109) for OUD diagnosis (OR, 3.009; 95% CI, 2.948–3.071), opioid poisoning (OR, 1.890; 95% CI, 1.802–1.981), and pain-related ED visits (OR, 1.204; 95% CI, 1.192–1.215), but lower odds for all-cause mortality (OR, 0.895; 95% CI, 0.886–0.904) and MOUD receipt (OR, 0.886; 95% CI, 0.841–0.932). Across programs, individuals with both types of identified LTOT (113,211 in Medicaid, 486,418 in Medicare) had the highest risk of OUD diagnosis and opioid poisoning.

**Conclusions and relevance:**

Diagnostic codes demonstrate low sensitivity (30.5–34.6%) and only fair agreement (κ = 0.210–0.313) with prescription-based LTOT, indicating these methods identify largely different populations. These distinct populations show divergent, and often reversed, risk profiles across Medicare and Medicaid. Reliance on a single LTOT method of identification risks systematic misclassification of opioid-related risk, and population-specific, multi-method approaches to LTOT surveillance are needed.

## Introduction

Opioid use disorder (OUD) remains a significant public health crisis in the United States, associated with substantial morbidity, mortality, and healthcare costs. [1–3] Long-term opioid therapy (LTOT) is associated with substantially increased risk of the development of OUD, with risk increasing by dose and duration of exposure. [4] Two primary methods identify LTOT in administrative data: prescription-based definitions, which represent the most common approach and require 90 days of continuous opioid supply, [5,6] and diagnostic code-based identification using the International Classification of Diseases, 10th Revision (ICD-10) Z79.891 code (long-term use of opiate analgesic). [5–12] Yet the relative effectiveness of these LTOT identification methods in predicting OUD and other opioid related adverse outcomes remains incompletely understood. [5,6,13,14] The stakes for accurate identification are considerable. Patients with long-term opioid exposure face risks including the development of OUD, opioid poisoning, overdose mortality, and other adverse health outcomes. [4,15–19] Effective risk stratification using appropriate LTOT identification methods could enable more targeted monitoring, support, and intervention. [15] Conversely, inaccurate identification might lead to mis-allocated resources, inadequate risk management, or potentially harmful stigmatization [20].

Each method has inherent limitations that may affect population identification. Prescription-based definitions in administrative databases such as Medicare and Medicaid fundamentally measure prescribing behavior, the duration or number of prescriptions over a defined period, rather than a clinician’s assessment of the patient’s chronic opioid use status or intent. [5,21] Prescription-based definitions also depend on pharmacy claims completeness and cannot capture opioids obtained through cash payments. As such, they may capture patients whose prescriptions do not reflect actual consumption patterns, miss patients whose opioid exposure is documented through other means, and are subject to variation in prescribing documentation across programs and settings. Code-based identification using Z79.891, by contrast, reflects a clinician’s diagnostic judgment that LTOT is clinically relevant, but depends on documentation behavior that varies across settings and may reflect clinical contexts beyond opioid exposure duration, such as care coordination, tapering monitoring, or risk documentation. [8] Despite widespread use of both approaches, no study has assessed the criterion validity of the Z79.891 diagnostic code against a prescription-based reference standard across complete national Medicare and Medicaid populations. Without understanding how well these definitions agree and where they diverge, it is impossible to interpret differences in associated risk profiles or to know whether studies using one method can be compared to those using the other.

The performance of LTOT identification methods may differ substantially between Medicare and Medicaid populations, which represent distinct demographic profiles and clinical contexts. [22–24] Medicare primarily serves adults aged 65 and older and people with disabilities, while Medicaid covers low-income individuals across the age spectrum. [25,26] These populations differ in their patterns of healthcare utilization, comorbidities, and potentially in how clinicians apply diagnostic codes and prescribe medications [27,28].

In this study, we first assessed the criterion validity of the Z79.891 diagnostic code for identifying LTOT using prescription-based LTOT as the reference standard across the complete Medicare and Medicaid populations. We then compared how these two identification methods correspond to the risk of OUD, opioid-related adverse outcomes, and treatment receipt, and explored how demographic and clinical characteristics modify these relationships. Understanding the agreement between, and differential risk associated with, these LTOT identification methods may inform more effective approaches to opioid risk surveillance and intervention.

## Methods

### Data sources and study approval

This study received approval from the Wake Forest University School of Medicine Institutional Review Board (IRB00085614) and the University of Rochester Research Subjects Review Board (RSRB) (STUDY00007315). We analyzed 100% Medicare Fee-For-Service (FFS), Medicare Advantage (MA), and Medicaid claims data spanning from January 1st, 2016, to December 31^st^, 2022 (earliest access date March 3rd, 2024, final access date March 24^th^, 2026). We ensured complete capture of dual-eligible (Medicare/Medicaid) individuals by linking their claims across both Medicare and Medicaid programs, which enabled identification of risk factors and outcomes across both programs while establishing a more precise index diagnosis and date. We analyzed the data inside the Chronic Conditions Warehouse (CCW) Virtual Research Data Center (VRDC) which contains Research Identifiable Files (RIFs) that were not fully anonymized. [29] All outputs and results went through CCW export review and were de-identified in accordance with the HIPAA Privacy rule which included aggregating results to the national cohort level, removing any individual identifiers, as well as removing cells with frequencies <11 that were small enough to facilitate re-identification of individuals.

### Cohort identification

We constructed three mutually exclusive cohorts to analyze population differences for different cases of LTOT coding. First, we identified incident diagnoses using two LTOT identification methods, prescription-based 90-day continuous opioid prescription criteria [5] and Z79.891 diagnostic code (S1 Fig). [7] We then applied exclusion restrictions: (i) index diagnosis to be between January 2017 and December 2020 (limiting index dates to 2017–2020 ensured that all individuals had the opportunity for a full two-year follow-up period within the 2022 data endpoint), (ii) no OUD diagnosis (e.g., F11.1*, F11.2*, F19.132, F19.139, F19.150) in the year prior to the index date, and (iii) no missing sex and at least one-year of continuous Medicare or Medicaid enrollment before and after the index date (unless they died within a year of the index event). Finally, individuals identified using both LTOT methods with index diagnoses within a year were analyzed as a separate group. This approach prioritized identifying an individual’s first opioid-related diagnosis to compare risks across similar timeframes. Once assigned to a cohort, individuals were not reclassified during follow-up, even if they subsequently met criteria for a different exposure category.

### Outcomes

Primary outcomes identified within 730 days following the index event included the presence of a documented OUD diagnosis code (requiring diagnoses on two different dates within the sample period as confirmation), the receipt of medications for OUD (MOUD) including buprenorphine, methadone, and naltrexone prescriptions identified through NDC/HCPCS codes, or opioid poisoning (T40* codes, excluding assault/adverse effect codes). [13,29] We also assessed all-cause mortality (2017–2022) and drug mortality (2017–2020 in Medicaid, 2017–2021 in Medicare), as well as emergency department visits for pain (see S1 File).

### Variable construction

Risk factors were indicated by any diagnosis in the year prior to the index event and included ICD-10 diagnostic codes for pain, anxiety, depression, bipolar disorder, abuse history, suicide attempt, tobacco use, stimulant use, and cannabis use disorders, and the full code list is provided (S1 File). [7,14,29] Demographic variables included age, sex, race, ethnicity, disability status (Medicaid), dual enrollment in Medicare and Medicaid, and Medicare Advantage enrollment. Index year fixed effects accounted for temporal changes in outcomes including COVID-19 pandemic patterns. Variables with missing values, including race and ethnicity, were handled using a complete-case approach with missingness treated as a separate analytic category; no imputation was performed.

### Coding and algorithms

Opioid use disorder diagnosis was identified using a modified Centers for Medicare and Medicaid (CMS) Chronic Conditions Warehouse (CCW) algorithm (Wake Forest-OUD) (see S1 File) [13,29] based on our previously published methodology requiring at least two diagnoses recorded on separate days within a 2-year period to improve specificity and reduce misclassification from rule-out diagnoses. [13,14,29–31] This timeframe extends beyond the DSM-5’s 12-month diagnostic criteria [32,33] to accommodate claims processing, healthcare utilization patterns in administrative data [29] and is consistent with previous recommendations. [13] Poisoning and MOUD definitions used Healthcare Common Procedure Coding System (HCPCS), Current Procedural Terminology (CPT), National Drug Code (NDC), and International Classification of Diseases, 10th Revision (ICD-10) codes described previously. [13,14,29] Buprenorphine National Drug Codes (NDCs) excluded formulations with a Food and Drug Administration (FDA) indication for pain. [34] Naltrexone MOUD was attributed to OUD only if no alcohol use disorder (AUD) was present or when both OUD and AUD diagnosis were coded. Methadone for OUD treatment was captured using HCPCS and CPT codes associated with opioid treatment programs (OTPs), as methadone for OUD is dispensed directly at certified OTPs rather than through retail pharmacies and therefore does not appear in standard pharmacy claims based on NDC. We grouped methadone NDCs dispensed through retail pharmacies for pain with other prescription opioids. We excluded laboratory-only and radiology-only claims for OUD diagnosis to align with prior literature identifying these as sources of overestimation, though we retained such claims for opioid poisoning outcomes. [8,13,14,35] ICD-10 code F11.9* (opioid use, unspecified) was explicitly excluded from the OUD codeset, as this code represents clinically documented opioid misuse rather than OUD per ICD-10 hierarchical guidance [7] and does not map to DSM-5 criteria for moderate or severe opioid use disorder [14,32,33].

### Criterion validity analysis

To assess the criterion validity of the Z79.891 diagnostic code, we constructed 2 × 2 contingency tables using the full beneficiary populations with any enrollment during the study period (N = 65.5 million Medicaid; N = 59.6 million Medicare). For each beneficiary, we determined (a) whether they met prescription-based LTOT criteria (≥90 days continuous opioid supply) at any point during the study period [5] (reference standard), (b) whether they had any Z79.891 diagnostic code during the same period (index test), and (c) whether they had index events for both criteria within one year. We calculated sensitivity, specificity, positive predictive value (PPV), negative predictive value (NPV), overall concordance, and Cohen’s kappa with 95% confidence intervals for this one-year agreement. Kappa values were interpreted using Landis and Koch benchmarks: < 0.00 poor, 0.00–0.20 slight, 0.21–0.40 fair, 0.41–0.60 moderate, 0.61–0.80 substantial, 0.81–1.00 almost perfect agreement [36].

### Statistical analysis

For the criterion validity analysis, we calculated sensitivity, specificity, PPV, NPV, observed agreement (concordance), expected agreement, and Cohen’s kappa using prescription-based LTOT as the reference standard, with 95% confidence intervals using Wilson score intervals for proportions and standard error estimation for kappa. For the risk-profile analysis, we employed logistic regression models to assess the association between cohort assignment and binary outcomes such as subsequent OUD diagnosis, poisoning, and MOUD initiation, while controlling for demographic and clinical covariates. We conducted stratified analyses by program (Medicare vs. Medicaid) to identify population-specific patterns. Our primary comparisons focused on the two LTOT identification methods (prescription-based vs. code-based) and their relative association with OUD treatment and opioid-related adverse outcomes. We did not apply formal corrections for multiple comparisons. Analyses were hypotheses-driven and focused on consistent patterns across outcomes and programs.

## Results

### Study population characteristics

The study population comprised 771,581 Medicaid beneficiaries and 4,376,993 Medicare beneficiaries (Table 1). Patients were identified through prescription-based LTOT (390,793 Medicaid, 1,045,466 Medicare), code-based LTOT (267,577 Medicaid, 2,845,109 Medicare), or both within a year (113,211 Medicaid, 486,418 Medicare). Medicaid beneficiaries were predominantly female (61.9%) and White (45.5%), with substantial proportions having pain diagnoses (87.7%) and mental health comorbidities including anxiety (31.7%) and depression (26.1%). The Medicare population was older (79.7% aged 65 and above), predominantly female (59.6%) and White (77.0%), with lower rates of pain diagnoses (77.1%) and mental health conditions but higher prevalence of tobacco use compared to the Medicaid cohort.

**Table 1:** Demographic and Clinical Characteristics of the Study Population Stratified by Identification Method and Insurance Type.

Variable	Medicaid Count	Percent of Total	Variable	Medicare Count	Percent of Total
**Medicaid Total**	771,581	100.00%	**Medicare Total**	4,376,993	100.00%
Diagnosis Cohort: Both	113,211	14.67%	Diagnosis Cohort: Both	486,418	11.11%
Diagnosis Cohort: 90 Day LTOT	390,793	50.65%	Diagnosis Cohort: 90 Day LTOT	1,045,466	23.89%
Diagnosis Cohort: Z79891	267,577	34.68%	Diagnosis Cohort: Z79891	2,845,109	65.00%
Risk Factor: Abuse	1,312	0.17%	Risk Factor: Abuse	3,852	0.09%
Risk Factor: Pain	677,000	87.74%	Risk Factor: Pain	3,373,025	77.06%
Risk Factor: Anxiety	244,794	31.73%	Risk Factor: Anxiety	1,167,707	26.68%
Risk Factor: Bipolar	80,520	10.44%	Risk Factor: Bipolar	213,811	4.88%
Risk Factor: Depression	201,220	26.08%	Risk Factor: Depression	1,160,866	26.52%
Risk Factor: Suicide	19,926	2.58%	Risk Factor: Suicide	41,095	0.94%
Risk Factor: COPC Pain	379,866	49.23%	Risk Factor: COPC Pain	1,830,597	41.82%
Risk Factor: Tobacco	222,410	28.83%	Risk Factor: Tobacco	1,556,144	35.55%
Risk Factor: Any Substance	241,132	31.25%	Risk Factor: Any Substance	1,568,922	35.84%
Risk Factor: Polysubstance	28,988	3.76%	Risk Factor: Polysubstance	57,055	1.30%
Age: 29 or less	100,294	13.00%	Age: 29 or less	11,550	0.26%
Age: 30–45	246,942	32.00%	Age: 30–45	120,631	2.76%
Age: 46–64	403,889	52.35%	Age: 46–64	756,503	17.28%
Age: 65–74	14,800	1.92%	Age: 65–74	1,696,552	38.76%
Age: 75+	5,656	0.73%	Age: 75+	1,791,757	40.94%
Sex: Female	477,459	61.88%	Gender: Female	2,607,131	59.56%
Sex: Male	294,122	38.12%	Gender: Male	1,769,862	40.44%
Race: American Indian	10,023	1.30%	Race/Ethnicity: American Indian	24,055	0.55%
Race: Asian	12,881	1.67%	Race/Ethnicity: Asian	65,660	1.50%
Race: Black	142,192	18.43%	Race/Ethnicity: Black	538,816	12.31%
Ethnicity: Hispanic	103,245	13.38%	Race/Ethnicity: Hispanic	313,252	7.16%
Race/Ethnicity: Multiracial/Other	151,855	19.68%	Race/Ethnicity: Multiracial/Other	66,893	1.53%
Race: White	351,385	45.54%	Race/Ethnicity: White	3,368,317	76.96%
SSDI: Yes	89,091	11.55%	Medicare Advantage Enrollment	1,747,875	39.93%
Dual Enrollment	71,154	9.22%	Dual Enrollment	1,312,681	29.99%
Start Year: 2017	285,031	36.94%	Start Year: 2017	1,385,977	31.67%
Start Year: 2018	201,539	26.12%	Start Year: 2018	1,135,417	25.94%
Start Year: 2019	151,956	19.69%	Start Year: 2019	997,581	22.79%
Start Year: 2020	133,055	17.24%	Start Year: 2020	858,018	19.60%
Mean CCI (SD)	1.18 (1.68)		Mean CCI (SD)	2.8 (2.42)	

*This table presents the demographic and clinical characteristics of Medicare and Medicaid beneficiaries identified through two different methods:* Z79.891 diagnostic code (long-term opioid therapy), and 90-day long-term opioid therapy based on prescription records or having both within a year. Characteristics include risk factors, age distribution, sex, race, ethnicity, disability status, dual-enrolled status, and index year.

### Criterion validity of Z79.891 against prescription-based LTOT

In the full Medicaid population with a pain diagnosis (N = 65.5 million), Z79.891 demonstrated sensitivity of 30.5% and specificity of 97.3% against the prescription-based LTOT reference standard. PPV was 40.2% and NPV was 95.9%. Overall concordance was 93.6%, but Cohen’s κ was 0.313, indicating only fair chance-corrected agreement (Table 2). In Medicare (N = 59.6 million), Z79.891 had sensitivity of 34.6%, specificity of 91.4%, PPV of 23.0%, and NPV of 95.0%. Concordance was 87.5% with Cohen’s κ of 0.210, indicating fair agreement.

**Table 2:** Criterion Validity Results for One-Year Agreement of Z79.891 Against Prescription-Based LTOT in Individuals Diagnosed with Pain in Medicare and Medicaid.

	Medicaid	Medicare
N	65,498,047	59,591,019
Rx LTOT Prevalence	5.6%	6.9%
Z79.891 Prevalence	4.2%	10.4%
Sensitivity	30.5% (30.4, 30.5)	34.6% (34.6, 34.6)
Specificity	97.3% (97.3, 97.3)	91.4% (91.4, 91.4)
PPV	40.2% (40.1, 40.3)	23.0% (22.9, 23.0)
NPV	95.9% (95.9, 95.9)	95.0% (94.9, 95.0)
Concordance	93.6% (93.6, 93.6)	87.5% (87.4, 87.5)
Kappa	0.313 (0.313, 0.314)	0.210 (0.210, 0.211)
Kappa Interpretation	Fair	Fair

Approximately two-thirds of prescription-based LTOT patients in both populations lacked Z79.891 coding (false negative rates of 69.5% in Medicaid and 65.4% in Medicare). Conversely, among patients coded with Z79.891, the majority did not meet prescription-based LTOT criteria (59.8% false discovery rate in Medicaid, 77.0% in Medicare). Medicare showed substantially more false positives (4.8 million vs. 1.7 million in Medicaid), contributing to its lower PPV and lower kappa. These findings establish that the two identification methods capture largely non-overlapping populations, providing context for the divergent risk profiles described below.

### Comparison between two LTOT identification methods

**Fig 1:**
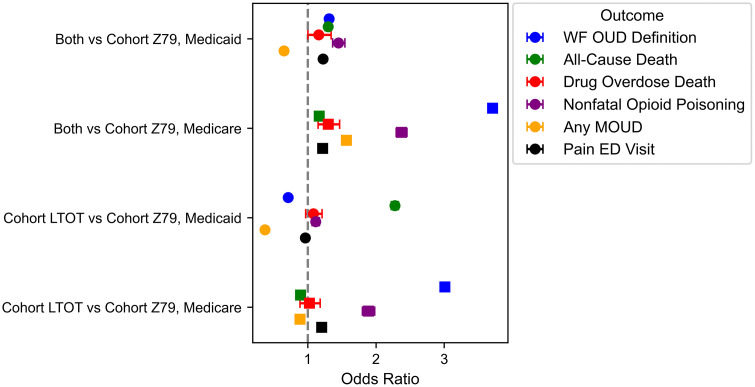
Comparative Odds of Clinical Outcomes Across LTOT Cohort Groups by Insurance Type.

Among Medicaid beneficiaries with LTOT (N = 771,581), the two LTOT identification methods demonstrated divergent risk profiles (Fig 1). Patients identified through the Z79.891 diagnostic code (n = 267,577) demonstrated higher risk for opioid-specific outcomes than those identified through prescription-based LTOT (n = 390,793) (Table 3). Compared to Z79.891 patients, the prescription-based LTOT group had significantly lower odds of receiving an OUD diagnosis (OR, 0.713; 95% CI, 0.696–0.730), pain-related ED visits (OR, 0.965; 95% CI, 0.955–0.975), or MOUD receipt (OR, 0.374; 95% CI, 0.364–0.385). However, these patients showed increased risk for all-cause mortality (OR, 2.277; 95% CI, 2.224–2.331) and opioid poisoning (OR, 1.117; 95% CI, 1.063–1.173), with similar risk for drug overdose death (OR, 1.083; 95% CI, 0.969–1.211) (Fig 1). The Charlson Comorbidity Index (CCI) was significantly lower for code-based LTOT patients (mean 1.014, 95% CI 1.008–1.020) than prescription-based LTOT patients (mean 1.286, 95% CI 1.280–1.291), suggesting the prescription-based LTOT method captured patients with greater overall medical complexity despite lower rates of opioid-specific adverse outcomes (S1 Table).

**Table 3:** Multivariate Logistic Regression Results for Opioid Use Disorder Diagnosis Outcome in Medicare and Medicaid.

Variable	Medicaid Estimate	Medicaid 95% CI	Medicare Estimate	Medicare 95% CI
Diagnosis Cohort: Both vs Cohort Z79	1.313	[1.276, 1.351]	3.710	[3.657, 3.764]
Diagnosis Cohort: Cohort LTOT vs Cohort Z79	0.713	[0.696, 0.730]	3.009	[2.948, 3.071]
Risk Factor: Abuse	0.887	[0.728, 1.080]	1.067	[0.944, 1.205]
Risk Factor: Pain	0.630	[0.610, 0.651]	1.242	[1.214, 1.272]
Risk Factor: Anxiety	1.259	[1.230, 1.289]	1.137	[1.120, 1.154]
Risk Factor: Bipolar	1.163	[1.127, 1.199]	1.142	[1.116, 1.168]
Risk Factor: Depression	1.073	[1.047, 1.099]	1.175	[1.157, 1.192]
Risk Factor: Suicide	1.132	[1.073, 1.195]	1.315	[1.259, 1.373]
Risk Factor: COPC Pain	1.055	[1.031, 1.079]	1.411	[1.391, 1.431]
Risk Factor: Tobacco	0.881	[0.834, 0.931]	0.721	[0.668, 0.777]
Risk Factor: Any Substance	1.698	[1.608, 1.792]	1.474	[1.367, 1.589]
Risk Factor: Polysubstance	1.458	[1.397, 1.523]	1.575	[1.521, 1.630]
Age: 29 or Less vs 30–45	0.855	[0.828, 0.882]	1.167	[1.082, 1.260]
Age: 46–64 vs 30–45	0.567	[0.554, 0.580]	0.731	[0.713, 0.750]
Age: 65–74 vs 30–45	0.541	[0.494, 0.592]	0.477	[0.465, 0.489]
Age: 75 + vs 30–45	0.140	[0.105, 0.186]	0.341	[0.332, 0.350]
Sex: Male vs Female	1.274	[1.248, 1.302]	1.095	[1.082, 1.108]
Race: American Indian vs White	1.032	[0.951, 1.119]	1.107	[1.032, 1.187]
Race: Asian vs White	0.407	[0.361, 0.459]	0.710	[0.669, 0.754]
Race: Black vs White	0.626	[0.607, 0.645]	0.869	[0.854, 0.885]
Ethnicity: Hispanic vs White	0.482	[0.463, 0.501]	1.128	[1.105, 1.151]
Race/Ethnicity: Multiracial/Other vs White	0.741	[0.721, 0.761]	0.917	[0.873, 0.963]
TAF: SSDI Enrollment, Medicare: MA Enrollment	0.914	[0.883, 0.947]	1.359	[1.343, 1.374]
Any Dual Enrollment in 2 Years	1.297	[1.251, 1.345]	1.127	[1.112, 1.142]
Start Year: 2018 vs 2017	0.856	[0.834, 0.878]	0.955	[0.941, 0.969]
Start Year: 2019 vs 2017	0.816	[0.793, 0.840]	0.913	[0.899, 0.928]
Start Year: 2020 vs 2017	0.791	[0.767, 0.815]	0.893	[0.878, 0.909]

Forest plot (Fig 1) showing odds ratios (ORs) and corresponding confidence intervals for multiple clinical outcomes comparing (1) patients meeting both Prescription and Code-based LTOT criteria versus Code-based alone, and (2) Prescription versus Code-based LTOT cohorts, stratified by Medicaid and Medicare populations. Outcomes include (Blue dot) Outcome by modified (Wake Forest) Chronic Conditions Warehouse (WF-OUD) code-based definition of Opioid Use Disorder (OUD). (Green dot) Outcome of All-Cause Mortality. (Red dot) Outcome of Opioid Overdose Mortality. (Purple dot) Outcome of Nonfatal Opioid Poisoning. (Yellow dot) Outcome of Use of Medications for Opioid Use Disorder (MOUD). (Black dot) Outcome of an Emergency Department visit for Pain. The vertical dashed line represents an OR of 1.0 (no difference). Points to the right of the line indicate increased odds, and points to the left indicate decreased odds relative to the reference group.

Individuals who had both diagnoses within a year (n = 113,211) had the highest OUD risks overall. Compared to individuals with just the Z79.891 code, individuals with both had higher risks of OUD diagnosis (OR, 1.313; 95% CI, 1.276–1.351), opioid poisoning (OR, 1.452; 95% CI, 1.365–1.545), drug-overdose death (OR, 1.160; 95% CI, 1.002–1.342), all-cause death (OR, 1.299; 95% CI, 1.255–1.344), and pain-related ED visit (OR, 1.224; 95% CI, 1.207–1.242). Those risks were also all statistically higher than individuals with just prescription-based LTOT, except for drug overdose death which was not significantly different, and all-cause mortality which was lower than prescription-based LTOT. Individuals with both diagnoses had lower rates of MOUD compared to individuals with just Z79.891 (OR, 0.652; 95% CI, 0.629–0.676), but higher MOUD rates than individuals with just prescription-based LTOT. The group with both diagnoses also had lower CCI than prescription-based LTOT and more than Z79.891 groups (mean 1.231, 95% CI 1.221–1.241).

Among Medicare beneficiaries with LTOT (N = 4,376,993), the risk pattern between the two LTOT identification methods was reversed. Patients identified through prescription-based LTOT (n = 1,045,466) consistently demonstrated higher risk for adverse outcomes compared to those identified through the code-based LTOT (n = 2,845,109) (Table 3). The prescription-based LTOT group had significantly higher odds of receiving an OUD diagnosis (OR, 3.009; 95% CI, 2.948–3.071), opioid poisoning (OR, 1.890; 95% CI, 1.802–1.981), and pain-related ED visits (OR, 1.204; 95% CI, 1.192–1.215). However, patients were less likely to receive MOUD (OR, 0.886; 95% CI, 0.841–0.932) or die from any cause (OR, 0.895; 95% CI, 0.886–0.904) with similar risk of drug overdose death (OR, 1.024; 95% CI, 0.886–1.182). Unlike Medicaid, the average CCI for Z79.891 patients (mean 2.850, 95% CI 2.847–2.852) was significantly higher than the prescription-based LTOT group (mean 2.741, 95% CI 2.736–2.746), and the group with both had the lowest overall CCI (mean 2.655, 95% CI 2.648–2.662).

Individuals who had both diagnoses within a year (n = 486,418) had the highest OUD risks overall. Compared to individuals with just the Z79.891 code, individuals with both had higher risks of OUD diagnosis (OR, 3.710; 95% CI, 3.657–3.764), opioid poisoning (OR, 2.375; 95% CI, 2.298–2.454), drug-overdose death (OR, 1.301; 95% CI, 1.153–1.468), all-cause death (OR, 1.170; 95% CI, 1.161–1.180), pain-related ED visit (OR, 1.218; 95% CI, 1.209–1.226), and MOUD (OR, 1.568; 95% CI, 1.516–1.621). Those risks were also all statistically higher than individuals with just prescription-based LTOT, except for drug overdose death and drug overdose which were not significantly different.

### Demographic and clinical risk factors

**Fig 2:**
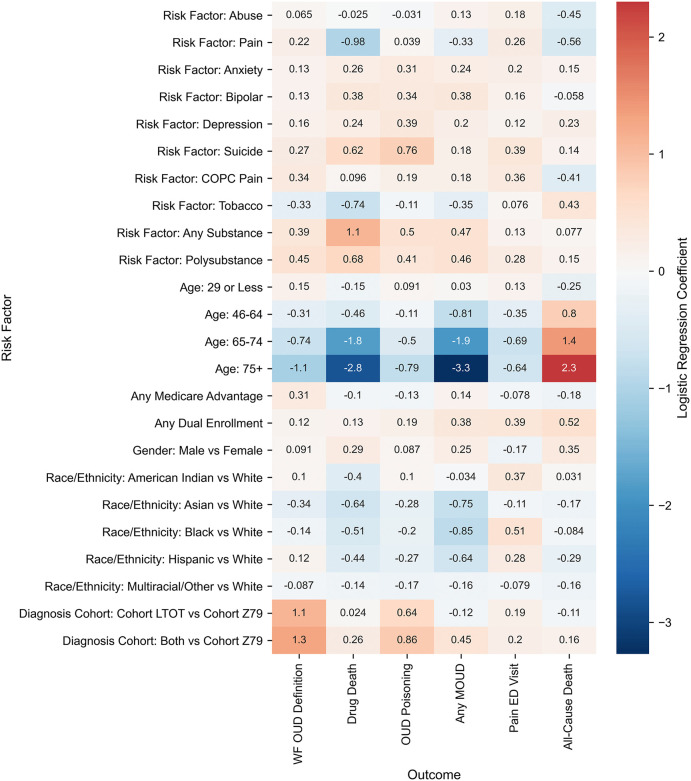
Heat map of adjusted log odds ratios (ORs) for clinical outcomes across LTOT identification methods (prescription-based, Z79.891, both) in Medicare. Outcomes include OUD, opioid poisoning, overdose mortality, all-cause mortality, MOUD, and pain-related ED visits within 2 years. Colors indicate relative log odds for that risk factor or identification method vs Z79.891 (reference).

**Fig 3:**
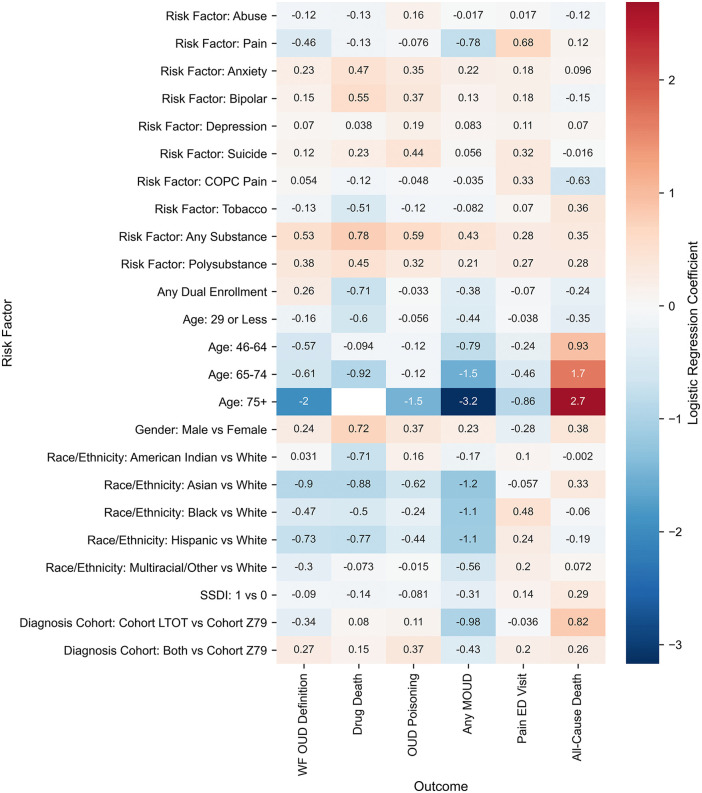
Heat map of adjusted log odds ratios (ORs) for clinical outcomes across LTOT identification methods (prescription-based, Z79.891, both) in Medicaid. Outcomes include OUD, opioid poisoning, overdose mortality, all-cause mortality, MOUD, and pain-related ED visits within 2 years. Colors indicate relative log odds for that risk factor or identification method vs Z79.891 (reference).

Multivariate logistic regression revealed consistent demographic patterns for OUD across both programs (Figs 2 and 3). Males had higher odds than females in Medicaid (OR, 1.274; 95% CI, 1.248–1.302) and Medicare (OR, 1.095; 95% CI, 1.082–1.108) (Table 3). OUD risk peaked at ages 30–45 in Medicaid, with progressive declines in older age groups (ages ≥75: OR, 0.140; 95% CI, 0.105–0.186). Medicare showed similar age gradients though less pronounced, with youngest beneficiaries (<29) showing 16.7% higher odds and oldest (≥75) showing 65.9% lower odds compared to ages 30–45.

Racial and ethnic patterns differed between programs. In Medicaid, American Indians showed similar odds to Whites (OR, 1.032; 95% CI, 0.951–1.119), while Asian (OR, 0.407; 95% CI, 0.361–0.459), Black (OR, 0.626; 95% CI, 0.607–0.645), Hispanic (OR, 0.482; 95% CI, 0.463–0.501), and multiracial or other groups (OR, 0.741; 95% CI, 0.721–0.761) demonstrated significantly lower odds. In Medicare, Hispanics (OR, 1.128; 95% CI, 1.105–1.151) and American Indians (OR, 1.107; 95% CI, 1.032–1.187) both had higher risks compared to Whites, while Asian (OR, 0.710; 95% CI, 0.669–0.754), Black (OR, 0.869; 95% CI, 0.854–0.885), and multiracial or other groups (OR, 0.917; 95% CI, 0.873–0.963) all had lower risks. Mental health comorbidities consistently increased OUD risk. In Medicaid: anxiety (OR, 1.259; 95% CI, 1.230–1.289), bipolar disorder (OR, 1.163; 95% CI, 1.127–1.199), depression (OR, 1.073; 95% CI, 1.047–1.099). Medicare showed similar patterns. Any substance use disorder significantly increased odds in both Medicaid (OR, 1.698; 95% CI, 1.608–1.792) and Medicare (OR, 1.474; 95% CI, 1.367–1.589). Non-opioid polysubstance use showed increased odds in Medicare (OR, 1.575; 95% CI, 1.521–1.630) and Medicaid (OR, 1.458; 95% CI, 1.397–1.523).

Pain-related diagnoses showed divergent patterns. In Medicaid, pain diagnosis was associated with lower OUD odds (OR, 0.630; 95% CI, 0.610–0.651) while the chronic overlapping pain conditions were associated with higher OUD odds (OR, 1.055; 95% CI, 1.031–1.079). In Medicare, both pain diagnosis (OR, 1.242; 95% CI, 1.214–1.272) and chronic overlapping pain conditions (OR, 1.411; 95% CI, 1.391–1.431) increased OUD odds.

### Treatment gaps

MOUD receipt rates were low across all groups (0.5% Medicare to 3.8% Medicaid), revealing treatment gaps transcending identification method.

## Discussion

### Principal findings

Our criterion validity analysis reveals that Z79.891 diagnostic code has low sensitivity (30.5% Medicaid, 34.6% Medicare) and only fair agreement (κ = 0.210–0.313) with prescription-based LTOT, establishing that these two widely used methods identify largely non-overlapping populations. This poor concordance provides the mechanistic explanation for our second key finding: the two methods correspond to strikingly divergent risk profiles for OUD and opioid-related adverse outcomes, with the direction of these associations reversing between Medicare and Medicaid. Code-based LTOT identified higher-risk populations than prescription-based LTOT in Medicaid for opioid-specific outcomes, while the opposite pattern emerged in Medicare. These findings confirm that the two LTOT identification methods are not interchangeable, and their operational meaning varies significantly across healthcare systems and populations.

### Interpreting the criterion validity findings

The low sensitivity and high specificity pattern indicate that Z79.891 functions as a confirmatory code: its presence signals clinician recognition of LTOT, but its absence does not exclude LTOT. This is consistent with the dependence on clinician documentation behavior rather than objective measurement. Notably, PPV was nearly twice as high in Medicaid (40.2%) compared to Medicare (23.0%), likely reflecting that Z79.891 is used more broadly in Medicare (prevalence 10.4% vs. 4.2% in Medicaid), perhaps for care coordination in older patients with polypharmacy, chronic pain monitoring, or tapering documentation contexts where the code serves a clinical purpose beyond confirming continuous 90-day opioid supply. The substantial false-positive population, patients coded with Z79.891 who do not meet prescription-based LTOT criteria, likely reflects several overlapping clinical scenarios rather than coding error. Clinicians may apply Z79.891 prospectively when initiating what they anticipate will become long-term therapy before the 90-day threshold is reached, retain the code for patients being actively tapered, or apply it to patients with episodic but recurrent opioid courses who are judged to be functionally on long-term therapy despite not satisfying a strict continuity definition. However, some coding may also reflect clinician use of Z79.891 as a clinical risk flag, documenting concern about a patient’s opioid use pattern rather than strictly indicating continuous exposure. [14] This interpretation is supported by the higher rates of OUD diagnosis and MOUD receipt among code-based LTOT patients in Medicaid, suggesting selective application to patients perceived as having problematic use – opioid misuse – effectively conflating long-term use documentation with risk identification. In Medicare, risk-adjustment incentives in Medicare Advantage plans may further encourage broader diagnostic documentation, consistent with the substantially higher Z79.891 prevalence and larger false-positive pool in that program. Taken together, these patterns indicate that a meaningful fraction of “false positives” represent a clinically recognized population whose opioid exposure or risk is real but not captured by pharmacy-claims algorithms, and whose risk profile differs from both prescription-based LTOT patients and unexposed beneficiaries.

These validity findings help explain the risk-profile reversal between payers. In Medicare, the large pool of Z79.891-coded patients who do not meet prescription-based LTOT criteria (4.8 million false positives) likely includes many patients with intermittent, lower-intensity, or recently discontinued opioid use, a lower-risk group that dilutes the risk signal for the code-based cohort. Meanwhile, prescription-based LTOT patients who lack Z79.891 coding may include those receiving prescriptions without active clinical monitoring, potentially representing a higher-risk subgroup. In Medicaid, the smaller false-positive pool and different coding context produces the opposite pattern. Cohen’s kappa of 0.313 (fair) in Medicaid and 0.210 (fair) in Medicare confirms that these definitions should not be treated as interchangeable in any research or policy context, with direct implications for interpreting the existing literature that may rely on one method without acknowledging that it captures a fundamentally different population than the other.

The low PPV of Z79.891 in Medicare (23.0%) has immediate policy implications. Nearly 4 in 5 patients flagged by this code do not have continuous 90-day opioid exposure by pharmacy records. Quality metrics and surveillance systems that rely on Z79.891 alone will substantially overcount LTOT in Medicare while simultaneously missing two-thirds of prescription-based LTOT patients. Conversely, prescription-based metrics alone will miss the clinical population that clinicians have recognized as warranting LTOT documentation. These findings suggest CMS and state agencies adopt dual-method surveillance.

### Policy implications for LTOT identification

Despite widespread use and recommendations for both LTOT identification methods, [5,8,14,37,38] this divergence has critical implications for research methodology and clinical practice. [3,14] Current federal quality metrics and state prescription drug monitoring programs rely exclusively on prescription-based LTOT identification methods, tracking pharmacy dispensing data rather than diagnostic codes. Our results suggest that such approaches may systematically misclassify risk depending on the insurance context. Studies that rely exclusively on one LTOT identification method may capture substantively different patient populations depending on insurance programs.

While prescription-based methods directly measure opioid exposure, diagnostic coding identifies patients for whom clinicians have recognized LTOT as clinically significant, potentially reflecting care coordination and monitoring. These complementary approaches capture different clinical contexts: prescription data measures drug exposure while diagnostic codes reflect clinical documentation, care oversight, and may identify patients receiving opioids through cash payments or medication outside insurance systems. Policymakers should develop insurance-specific risk stratification algorithms that account for the differential performance of LTOT identification methods, and quality measurement systems should employ multiple surveillance approaches using both prescription-based criteria and diagnostic codes. The Z79.891 code may function differently across programs, potentially capturing patients undergoing tapering or risk monitoring, necessitating clarified coding guidance and standardized application [8].

### Addressing treatment gaps

The MOUD receipt rates (0.5%–3.8%) in this study are substantially lower than the 8–12% estimated incidence of OUD among patients with chronic pain on LTOT, [39] suggesting systemic barriers that transcend LTOT identification method. [40,41] Low rates among older adults may reflect systemic barriers including clinician stigma, lack of training, limited access to opioid treatment programs, and insufficient integration of age-friendly care with substance use treatment. [42,43] Age-specific barriers were compounded by broader systemic obstacles during the study period, such as absent Medicare methadone reimbursement prior to 2020. [44] While Medicaid generally had coverage for MOUD before 2020, [45] access remains uneven due to clinician shortages, stigma, utilization management practices, and state-level variation in covered services. [46,47] Recent policy changes expanding methadone access in Medicare represent important progress, but comprehensive reform requires addressing workforce capacity, reimbursement adequacy, and integration of substance use treatment across care settings.

### Clinical and research implications

For clinical practice, these findings underscore the need for multi-modal risk assessment rather than reliance on single LTOT identification methods. Clinical decision support tools should incorporate both prescription-based monitoring and diagnostic coding for LTOT, [3,14] For research, these findings challenge the generalizability of studies using single LTOT identification methods. Substantial heterogeneity exists in LTOT definitions across the literature, [5] with many studies relying on diagnostic codes without prescription-based verification despite evidence that code-based identification alone captures only approximately half of clinically confirmed OUD cases. [31] Researchers should exercise caution when applying findings from one insurance population to another and consider population-specific validation. Future research and quality improvement initiatives should employ multiple identification approaches, including both LTOT methods, to ensure comprehensive risk assessment and intervention targeting.

### Understanding the medicaid-medicare divergence in LTOT methods

Several factors may explain why the two LTOT identification methods show divergent risk patterns across programs. In Medicaid, prescription-based LTOT may capture patients with greater overall medical complexity (mean CCI 1.286 vs 1.014 for Z79.891) who receive more structured healthcare oversight, potentially reducing other opioid related adverse outcomes risk despite greater opioid exposure. [48] In Medicare, the pattern reverses (CCI 2.741 for prescription-based LTOT vs 2.850 for code-based), and the Z79.891 diagnostic code may be applied in diverse clinical scenarios including patients with stable, monitored LTOT, those undergoing tapering, or as part of risk monitoring protocols [8].

This finding challenges simplistic narratives about prescription opioid exposure and risk, indicating that relationships between LTOT and adverse outcomes are mediated by patient characteristics, clinical context, and how LTOT is identified. [49] The two LTOT identification methods appear to capture partially overlapping but distinct patient populations with varying risk profiles, and these populations differ systematically between insurance programs [7,14,50,51].

### Demographic and clinical risk modifiers

Age patterns showed higher risk concentrated in younger beneficiaries in both programs, potentially reflecting survivor effects, cohort differences in substance use patterns, or differences in opioid prescribing practices for older adults. [52,53] The consistent identification of mental health conditions as OUD risk factors aligns with extensive literature on the intersection of mental health and substance use disorders. [54,55] Significantly higher odds for chronic overlapping pain conditions versus single anatomical site pain in Medicare highlights pain management complexity and its relationship to opioid-related outcomes. [56,57] This risk is amplified by central sensitization mechanisms, additive effects of multi-site pain, and comorbid mental health conditions that drive higher rates of LTOT despite limited efficacy and elevated adverse event risks [57,58].

Analysis across programs revealed significant dual enrollment effects. In Medicare, dual status associated with increased odds for all adverse outcomes including OUD (OR, 1.127; 95% CI, 1.112–1.142), opioid poisoning (OR, 1.209; 95% CI, 1.173–1.246), ED visit for pain (OR, 1.474; 95% CI, 1.466–1.482) and all-cause death (OR, 1.688; 95% CI, 1.679–1.698). However, in Medicaid, dual status associated with increased OUD odds (OR, 1.297; 95% CI, 1.251–1.345) but reduced odds for poisoning, pain ED visit, and mortality outcomes. This pattern indicates that dual status in Medicare is associated with relatively more health complications than dual status in Medicaid, consistent with previous work during the opioid prescribing epidemic [53].

### Interpreting the disconnect between mortality outcomes

The disconnect between opioid-specific mortality and all-cause mortality warrants attention. [59] In Medicaid, patients identified through prescription-based LTOT had similar odds of opioid overdose mortality but substantially higher all-cause mortality versus code-based LTOT patients. Medicare showed the reverse pattern. These patterns appear consistent with the two LTOT identification methods capturing patients with different overall medical complexity profiles, where greater comorbidity burden may paradoxically associate with more structured healthcare oversight and monitoring.

### Strengths and limitations

This study leveraged 100% Medicare and Medicaid claims data, providing comprehensive national coverage and eliminating sampling bias. The large sample size enabled detection of clinically meaningful differences across multiple outcomes and direct comparison of two LTOT identification methods. However, administrative claims cannot capture prescriptions obtained outside these insurance systems, potentially leading to misclassification. The observational design precludes causal inference, and residual confounding from unmeasured factors such as pain severity and functional status may influence observed associations.

Coding practices likely vary across healthcare systems and geographic regions, introducing heterogeneity in how diagnostic codes are applied. The Z79.891 code may be used inconsistently, for ongoing LTOT monitoring, during tapering, or for risk documentation, and we cannot fully distinguish these contexts from claims data alone. Generalizability beyond Medicare and Medicaid populations remains unknown. Temporal relationships between diagnoses and outcomes may involve bidirectional causality, as clinicians may apply diagnostic codes retrospectively after adverse events occur. The 2-year follow-up period may be insufficient to capture long-term consequences although it aligns with evidence that most opioid-related adverse events, including OUD diagnosis and overdose, occur within the first year to two years of opioid initiation, with risk particularly elevated in the first 60 days and increasing substantially after 90 days of cumulative use. [4,60] Sensitivity analyses examining alternative follow-up durations or alternative exposure definitions were not performed and represent an important area for future research.

Immortal time bias (ITB) is a potential concern in this analysis because the prescription-based LTOT definition requires accumulation of 90 days of opioid supply before cohort entry, whereas code-based entry occurs at first Z79.891 documentation. To mitigate this, the risk window for all cohorts began at the qualifying index date rather than at first opioid prescription, and patients who died or experienced outcomes before meeting the 90-day threshold were not included in the prescription-based LTOT cohort. Residual ITB may still affect comparisons between the two methods; however, ITB would be expected to make prescription-based LTOT appear at lower risk by excluding early adverse events. The direction of observed differences in Medicare where prescription-based LTOT patients demonstrated substantially higher risk for OUD diagnosis and opioid poisoning is therefore inconsistent with ITB-driven overestimation and suggests that any residual bias likely underestimates rather than overestimates the true magnitude of these risk differences. Caution should be applied when drawing causal inferences from comparisons across LTOT identification methods.

Methadone for OUD is dispensed exclusively through federally certified opioid treatment programs and is not captured in standard retail pharmacy (NDC) claims. Although we captured OTP-dispensed methadone through HCPCS and CPT codes, some OTP encounters may be incompletely recorded, potentially underestimating MOUD receipt among patients receiving methadone maintenance treatment.

Several additional limitations pertain to the criterion validity analysis. The prescription-based reference standard itself has limitations: it cannot capture opioids obtained outside insurance systems (e.g., cash payments), and some “false positives” for Z79.891 may therefore represent true LTOT patients not captured by pharmacy claims, meaning the true sensitivity of Z79.891 may be somewhat lower and the true PPV somewhat higher than reported. The criterion validity analysis classified beneficiaries based on whether their initial Z79.891 code and 90-day prescription window period occurred within a year of each other. This agreement increases with a larger co-occurrence window, but larger gaps may indicate changes over time in LTOT instead of diagnosis concordance. Supporting Information (S2 Table) shows the average time between prescription-based LTOT and the Z79.891 index dates for individuals who had both diagnoses in the full Medicare and Medicaid populations, and the timing is different based on which diagnosis is first. For individuals who first have prescription-based LTOT, 34% go on to receive a Z79.891 diagnosis (mean 596 days, median 420 days), but only 17% of individuals who first present with Z79.891 later receive 90-day LTOT (mean 311 days, median 122 days). Supporting Information (S3 Table) shows the criterion validity statistics allowing for two years difference between the two index diagnoses instead of one year. Medicare Advantage plans may have different incentive structures for diagnostic coding through risk adjustment compared to fee-for-service Medicare or Medicaid, which could partially explain payer differences in Z79.891 prevalence and validity metrics. Finally, Cohen’s kappa is influenced by prevalence, and with low overall LTOT prevalence in both populations, the kappa values may underestimate the true level of agreement relative to what would be observed in a higher-prevalence subpopulation.

## Conclusions

Diagnostic codes demonstrate poor criterion validity against prescription-based LTOT (sensitivity 30.5–34.6%, κ = 0.210–0.313), with the two methods capturing largely non-overlapping populations across Medicare and Medicaid. These distinct populations show divergent and often reversed risk profiles for OUD and opioid-related adverse outcomes between insurance programs, confirming that the definitions are not interchangeable. The code-based definition identified higher-risk Medicaid patients, while prescription-based criteria identified greater MOUD use and higher risk for OUD and adverse outcomes in Medicare. [22,23] Opioid risk surveillance, clinical decision support, and research using LTOT as an exposure or outcome should employ multiple identification methods calibrated to specific insurance contexts rather than applying universal algorithms. [1,28,61] The criterion validity data reported here provide a foundation for researchers to interpret existing literature and design future studies that account for definition-dependent ascertainment bias.

## Supporting information

S1 Fig Cohort attrition diagram. (DOCX)

S1 File Claims_Codes. (XLSX)

S1 Table Charlson Comorbidity Index (CCI) by Cohort, Dual-Status, and Program. (DOCX)

S2 Table Diagnosis Transition Timing for Prescription-Based LTOT, Z79.891, and OUD Across the Full Medicare and Medicaid Populations. (DOCX)

S3 Table Criterion Validity Results for Two-Year Agreement of Z79.891 Against Prescription-Based LTOT in Individuals Diagnosed with Pain in Medicare and Medicaid. (DOCX)
