# Supplementary figures and images for: Criterion validity and divergent risk profiles of long-term opioid therapy across medicare and medicaid
Source: PLoS One. 2026 Apr 29;21(4):e0347943. doi: 10.1371/journal.pone.0347943 (PMC13127924; doi:10.1371/journal.pone.0347943)

**S1 Figure: Cohort Attrition Diagram**


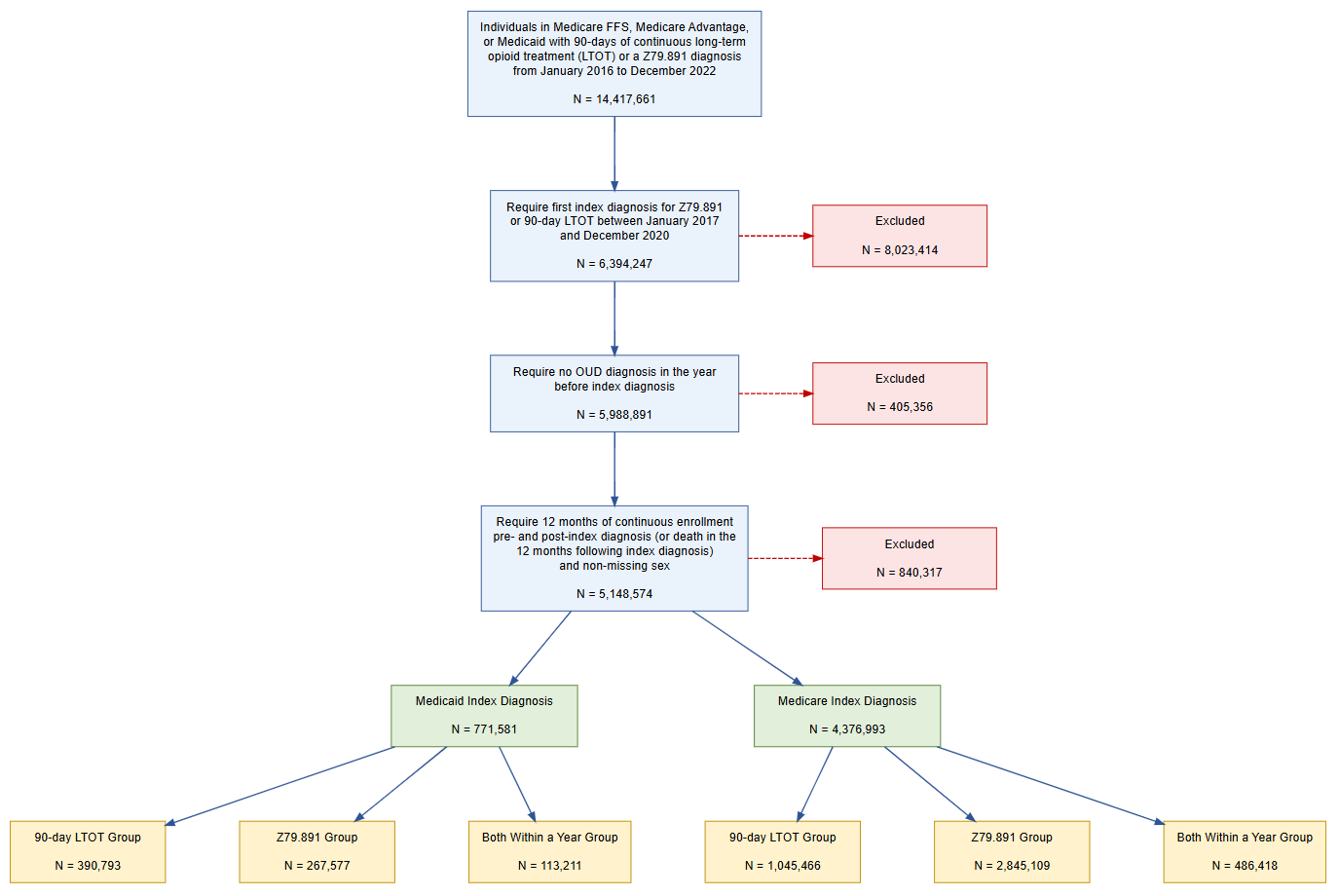

Supplement: S1 Fig — (DOCX) [file pone.0347943.s001.docx]
