# Supplementary material for: Criterion validity and divergent risk profiles of long-term opioid therapy across medicare and medicaid
Source: PLoS One. 2026 Apr 29;21(4):e0347943. doi: 10.1371/journal.pone.0347943 (PMC13127924; doi:10.1371/journal.pone.0347943)
Supplement: S1 Table — (DOCX) [file pone.0347943.s003.docx]

**S3 Table: Charlson Comorbidity Index (CCI) by Cohort, Dual-Status, and Program**

| Program (Medicare/Medicaid) | Final Cohort | Cohort Size (N) | Mean CCI | SD (CCI) |
| --- | --- | --- | --- | --- |
| Medicaid | Rx-Based LTOT | 390793 | 1.29 | 1.75 |
|  | Both | 113211 | 1.23 | 1.65 |
|  | Z79.891 | 267577 | 1.01 | 1.57 |
|  |  |  |  |  |
| Program (Medicare/Medicaid) | Final Cohort | Cohort Size (N) | Mean CCI | SD (CCI) |
| Medicare | Rx-Based LTOT | 1045466 | 2.74 | 2.37 |
|  | Both | 486418 | 2.66 | 2.37 |
|  | Z79.891 | 2845109 | 2.85 | 2.44 |

| Dual-Eligibility | Program (Medicare/Medicaid) | Cohort Size (N) | Mean CCI | SD (CCI) |
| --- | --- | --- | --- | --- |
| No | Medicaid | 700427 | 1.12 | 1.64 |
| No | Medicare | 3064312 | 2.59 | 2.3 |
| Yes | Medicaid | 71154 | 1.79 | 1.97 |
| Yes | Medicare | 1312681 | 3.3 | 2.61 |
