# Supplementary material for: Criterion validity and divergent risk profiles of long-term opioid therapy across medicare and medicaid
Source: PLoS One. 2026 Apr 29;21(4):e0347943. doi: 10.1371/journal.pone.0347943 (PMC13127924; doi:10.1371/journal.pone.0347943)
Supplement: S2 Table — (DOCX) [file pone.0347943.s004.docx]

**S4 Table: Diagnosis Transition Timing for Prescription-Based LTOT, Z79.891, and OUD Across the Full Medicare and Medicaid Populations**

| Prior Diagnosis | Later Diagnosis | Patients Progressed | Patients with Prior Diagnosis | Fraction of Patients Progressed | Mean Days Between Diagnoses | Median Days Between Diagnoses |
| --- | --- | --- | --- | --- | --- | --- |
| Rx-Based LTOT | Z79.891 | 2874784 | 8370247 | 0.343 | 596 | 420 |
| Rx-Based LTOT | OUD | 1586406 | 8370247 | 0.190 | 658 | 479 |
| Z79.891 | Rx-Based LTOT | 1820263 | 10742461 | 0.169 | 311 | 122 |
| Z79.891 | OUD | 1413341 | 10742461 | 0.132 | 523 | 321 |
| OUD | Rx-Based LTOT | 577856 | 5735683 | 0.101 | 307 | 120 |
| OUD | Z79.891 | 959514 | 5735683 | 0.167 | 548 | 360 |
