# Supplementary material for: Criterion validity and divergent risk profiles of long-term opioid therapy across medicare and medicaid
Source: PLoS One. 2026 Apr 29;21(4):e0347943. doi: 10.1371/journal.pone.0347943 (PMC13127924; doi:10.1371/journal.pone.0347943)
Supplement: S3 Table — (DOCX) [file pone.0347943.s005.docx]

**S5 Table: Criterion Validity Results for Two-Year Agreement of Z79.891 Against Prescription-Based LTOT in Individuals Diagnosed with Pain in Medicare and Medicaid.**

|  | Medicaid 2-Year Agreement | Medicare 2-Year Agreement |
| --- | --- | --- |
| N | 65,498,047 | 59,591,019 |
| Rx LTOT Prevalence | 5.7% | 7.2% |
| Z79.891 Prevalence | 4.7% | 11.0% |
| Sensitivity | 39.8% (39.7, 39.8) | 45.2% (45.2, 45.3) |
| Specificity | 97.4% (97.4, 97.4) | 91.6% (91.6, 91.6) |
| PPV | 48.6% (48.6, 48.7) | 29.5% (29.5, 29.5) |
| NPV | 96.4% (96.4, 96.4) | 95.6% (95.6, 95.6) |
| Concordance | 94.1% (94.1, 94.1) | 88.3% (88.3, 88.3) |
| Kappa | 0.407 (0.406, 0.408) | 0.296 (0.295, 0.296) |
| Kappa Interpretation | Moderate | Fair |
